# Impact of skeletal muscle loss during conversion therapy on clinical outcomes in lavage cytology positive patients with gastric cancer

**DOI:** 10.3389/fonc.2022.949511

**Published:** 2022-09-23

**Authors:** Ping’an Ding, Peigang Yang, Li Yang, Chenyu Sun, Shuya Chen, Min Li, Scott Lowe, Honghai Guo, Yuan Tian, Yang Liu, Qun Zhao

**Affiliations:** ^1^ The Third Department of Surgery, The Fourth Hospital of Hebei Medical University, Shijiazhuang, China; ^2^ Hebei Key Laboratory of Precision Diagnosis and Comprehensive Treatment of Gastric Cancer, Shijiazhuang, China; ^3^ The Department of Computed Tomography (CT)/Magnetic Resonance Imaging (MRI), The Fourth Hospital of Hebei Medical University, Shijiazhuang, China; ^4^ AMITA Health Saint Joseph Hospital Chicago, Chicago, IL, United States; ^5^ Newham University Hospital, London, United Kingdom; ^6^ College of Osteopathic Medicine, Kansas City University, Kansas City, MO, United States

**Keywords:** abdominal lavage cytology positive, gastric cancer, skeletal muscle loss, sarcopenia, conversion therapy, significant muscle loss

## Abstract

**Background:**

The relationship between sarcopenia and clinical outcomes during conversion therapy in patients with lavage cytology positive gastric cancer (GC-CY_1_) remains unclear. This study aimed to investigate the impact of sarcopenia and skeletal muscle loss on the efficacy of conversion therapy, tumour response and survival in GC-CY_1_ patients.

**Methods:**

Retrospective analysis of data from a prospective trial of conversion therapy conducted between April 2018 and August 2019 in patients with GC-CY_1_ (NCT03718624). Skeletal muscle index (SMI) was measured at the level of the third lumbar (L3) vertebra and the sarcopenia was defined using published cut-off points in all patients. We defined ΔSMI (%)/50 days above 9.53% for men and ΔSMI (%)/50 days above 8.81% for women as significant muscle loss (SML) and analysed the changes in skeletal muscle during conversion therapy in relation to treatment efficacy, survival and tumour response.

**Results:**

Of the 36 patients, 7 patients (19.44%) developed sarcopenia before conversion therapy, 6 (16.67%) developed new sarcopenia after conversion therapy, and 8 (22.22%) developed SML during treatment. Multivariate analysis showed that sarcopenia before treatment [Odds Ratio (OR) =8.923, 95%CI: 1.341-25.321, p=0.002] and SML during treatment (OR=7.803, 95%CI: 1.106-16.189, p=0.001) had a negative impact on the success rate of conversion therapy. Cox multifactorial analysis found that pre-treatment sarcopenia [overall survival (OS): Hazard Ratio (HR) =6.341, 95%CI: 1.269-18.943, p=0.001; progression-free survival (PFS): HR=8.212, 95%CI: 1.569-36.582, p=0.001], newly developed sarcopenia after conversion therapy (OS: HR=3.189, 95%CI: 1.023-9.811, p=0.012; PFS: HR=3.084, 95%CI: 1.042-14.236, p=0.013) and the presence of SML during treatment (OS: HR=10.234, 95%CI: 2.532-54.231, p=0.002; PFS: HR=9.562, 95%CI: 2.341-38.092, p=0.002) were independent risk factor for OS and PFS in GC-CY_1_ patients.

**Conclusion:**

Pre-treatment sarcopenia and the presence of SML during treatment are strongly correlated with the immediate and long-term outcomes of GC-CY_1_ patients and can be used as imaging markers to predict the treatment efficacy and prognosis of patients in clinical practice.

## Introduction

In recent years, neoadjuvant intraperitoneal and systemic (NIPS) paclitaxel is the mainstay of treatment for GC-CY_1_ patients and is widely used in clinical practice (1, 2). Our previous prospective study also found that NIPS paclitaxel in combination with apatinib had a higher conversion success rate than conventional intravenous chemotherapy, which could improve the prognosis of GC-CY_1_ patients (3). However, the gastrointestinal toxicity induced by chemotherapy during conversion therapy may lead to malnutrition and weight loss in these patients. Moreover, the majority of GC-CY_1_ patients are malnourished at the time of their initial visit, and gastrointestinal adverse reactions during conversion therapy will further aggravate malnutrition.

Recent evidence suggests that the deterioration in nutritional status and weight loss in patients with gastric cancer will be directly reflected in the loss of skeletal muscle, which will further lead to sarcopenia (4–6). Up till now, numerous studies have demonstrated that sarcopenia is a predictor of poor prognosis for surgical complications and overall survival (OS) in patients with a variety of solid tumours (7–9). Otherwise, emerging evidence suggests that sarcopenia is also associated with poor prognosis and increased treatment-related toxicity in patients with advanced gastric cancer (10, 11). Nevertheless, there are rare reports of studies on the potential prognostic impact of sarcopenia during conversion therapy in GC-CY_1_ patients.

Abdominal computed tomography (CT) imaging is now widely used to assess the clinical staging of GC-CY_1_ patients with gastric cancer before and after conversion therapy (12, 13). The diagnosis of sarcopenia can be easily obtained from skeletal muscle measurements at the level of the L3 lumbar spine and several studies have confirmed that CT is the ‘gold standard’ for measuring muscle area (14, 15). Hence, changes in skeletal muscle area measured by pre- and post-treatment CT images during conversion therapy in GC-CY_1_ patients can be used as a potential imaging marker to predict immediate and long-term clinical outcomes. Previous studies on the prognosis of GC-CY_1_ patients have focused on the optimisation of treatment regimens and the specificity of the tumour (16, 17). However, there is still a lack of evidence regarding the impact of sarcopenic status before and after conversion therapy and changes in skeletal muscle during therapy on clinical outcomes in GC-CY_1_ patients.

Therefore, our study aimed to retrospectively analyse GC-CY_1_ patients included in a prospective clinical trial (NCT03718624) to assess the impact of sarcopenia on the immediate and long-term clinical outcomes of GC-CY_1_ patients receiving NIPS paclitaxel in combination with apatinib conversion therapy by analysing the changes in skeletal muscle area at L3 level during treatment.

## Materials and methods

### Study design and participants

This study retrospectively analysed data from a prospective clinical study (NCT03718624) of 36 GC-CY_1_ patients who received NIPS paclitaxel in combination with apatinib conversion therapy from April 2018 to August 2019 at the Fourth Hospital of Hebei Medical University. The inclusion criteria were as follows: (1) all patients had gastric cancer confirmed histopathologically by gastroscopic biopsy and the human epidermal growth factor receptor 2 (HER2) tests were negative before the operation; (2) laparoscopic exploration and abdominal exfoliative cytology confirmed the presence of free cancer cells in the peritoneal cavity with the absence of peritoneal and distant organs metastases before conversion therapy; (3) age between 18 and 75 years; (4) organs with reserve functions were able to tolerate 3 cycles of NIPS paclitaxel and apatinib conversion therapy; (5) all patients underwent a repeat laparoscopic exploratory staging procedure after conversion therapy; (6) availability of complete hospitalisation data, including CT scans and follow-up data before and after conversion therapy. The exclusion criteria were as follows: (1) the coexistence of other malignancies; (2) organs with poor reserve function unable to tolerate surgery or patient refusal of surgical treatment, or unable to cooperate with treatment; (3) metal implants in the lumbar spine. This study was reviewed and approved by the ethics committee of the Fourth Hospital of Hebei Medical University (Ethics number: 2018088). All patients provided informed consent.

### Conversion therapy

All patients underwent laparoscopic exploration and abdominal exfoliation cytology first, patients with intraoperative pathologically confirmed free cancer cells in the abdominal cavity were treated with NIPS paclitaxel in combination with apatinib. The detailed conversion treatment protocol was reported in the previous study (3). All patients were followed up with laparoscopic exploration and intra-abdominal free cancer detection after 3 cycles of conversion therapy. Patients were treated with the original regimen and radical surgical resection was performed if no free cancer cells (FCC) in abdominal cavity, otherwise, conversion therapy was continued.

### Measurement and definition of body composition

We retrospectively analysed the CT images of all enrolled patients before and after conversion therapy. Previous studies have confirmed that the area of skeletal muscle and adipose tissue in CT cross-sectional images at the level of the L3 vertebral in the supine position correlates closely with whole-body muscle and fat mass (14, 15). Therefore, we chose to measure skeletal muscle area at the L3 vertebral level in patients with GC-CY_1_.

The 5-mm flat-scan images were uploaded to the Picture Archiving and Communication System (PACS, SIEMENS SOMATOM) after completing all image acquisition. Semi-automatic segmentation was performed by a board-certified radiologist (experience 5 years) and boundaries of the L3 vertebrae body were outlined along the inner edge of the abdominal wall subcutaneous fat. The Hounsfield unit (HU) threshold value was applied to skeletal muscle: attenuation range of -29 to 150 HU (18). The software evaluated and measured the pixel area of the corresponding skeletal muscle attenuation to obtain the skeletal muscle area (SMA). The cross-sectional area of skeletal muscle was divided by the square of height to obtain the skeletal muscle index (SMI), and all results were expressed in square meters (cm^2^/m^2^) and standardised for comparison.

Patients included in this study were Asian, so sarcopenia was defined using the results of Fujiwara et al. and Mardian et al. studies, which were conducted in Asian populations. Patients with SMI <36.2 cm^2^/m^2^ for men and SMI < 29.6 cm^2^/m^2^ for women were regarded as the presence of sarcopenia (19, 20). In addition, given the varying duration of conversion therapy with NIPS paclitaxel combined with apatinib in this cohort of patients, a standardised and uniform unit was used for comparison between groups. The median conversion treatment interval for the whole group of patients was 58 days. Therefore, the amount of change in SMI was calculated before and after conversion therapy[ΔSMI], the percentage of this value relative to the SMI before conversion therapy was recorded as ΔSMI [%] and it was then divided by the number of days apart and multiplied by 50 to represent the relative change over 50 days [ΔSMI[%]/50 days].

### Outcome measures

The Response Evaluation Criteria for Solid Tumours (RECIST) version 1.1 was used to evaluate imaging outcomes after conversion therapy (21), including complete remission (CR), partial response (PR), stable disease (SD) and progressive disease (PD). The percentage of patients with CR and PR is defined as the objective response rate (ORR), while the percentage of patients other than those with PD is defined as the preoperative disease control rate (DCR).

The pathological response is assessed and graded according to the TRG grading scale (AJCC/CAP criteria) using surgically resected specimens after conversion therapy (22). A TRG grade 0 is defined as no residual tumour cells, while sparse residual tumour cells can be seen microscopically as TRG grade 1, and more fibrosis than residual tumour cells as TRG grade 2. Conversely, a grade of TRG 3 is defined as having more residual tumour cells than fibrosis or no regressive changes. In this study, TRG grade 0 and TRG grade 1 were defined as major pathologic responses (MPR).

Meanwhile, the other endpoint observed in this study was overall survival (OS) and progression-free survival (PFS) at 2 years. OS was defined as the time interval from the initial date of conversion therapy to the date of last follow-up or death. In contrast, PFS was defined as the period from the start of conversion therapy to the date of disease progression, death or last follow-up, whichever occurred first. All patients were followed up every 3 months after starting treatment. In the meanwhile, enhanced CT scans were performed every 3 months for the first 3 years after surgery and then every 6 months for the next 2 years.

### Statistical analysis

SPSS version 21.0 and GraphPad Prism 8.01 were used for statistical analyses. In this study, all continuous data median (interquartile spacing) or mean ± standard deviation were expressed and analysed for comparison using an independent *t* test or Mann-Whitney U-test. The categorical data were expressed as numbers and percentages and analysed using the chi-square test or Fisher’s exact test. The optimal cut-off value for ΔSMI (%)/50 days was used to classify patients with good or poor OS. The optimal cut-off value for ΔSMI (%)/50 days was obtained using the receiver operating characteristic (ROC) curve for the highest Youden’s index. Survival curves were plotted using the Kaplan-Meier method, and the log-rank test was used to compare survival rates between groups. Univariate and multivariate analyses were performed using Cox proportional risk regression models, and results were shown as hazard ratio (HR) with 95% confidence intervals (CI). A p < 0.05 was considered statistically significant.

## Results

### Changes in sarcopenia during neoadjuvant therapy

**Figure 1:**
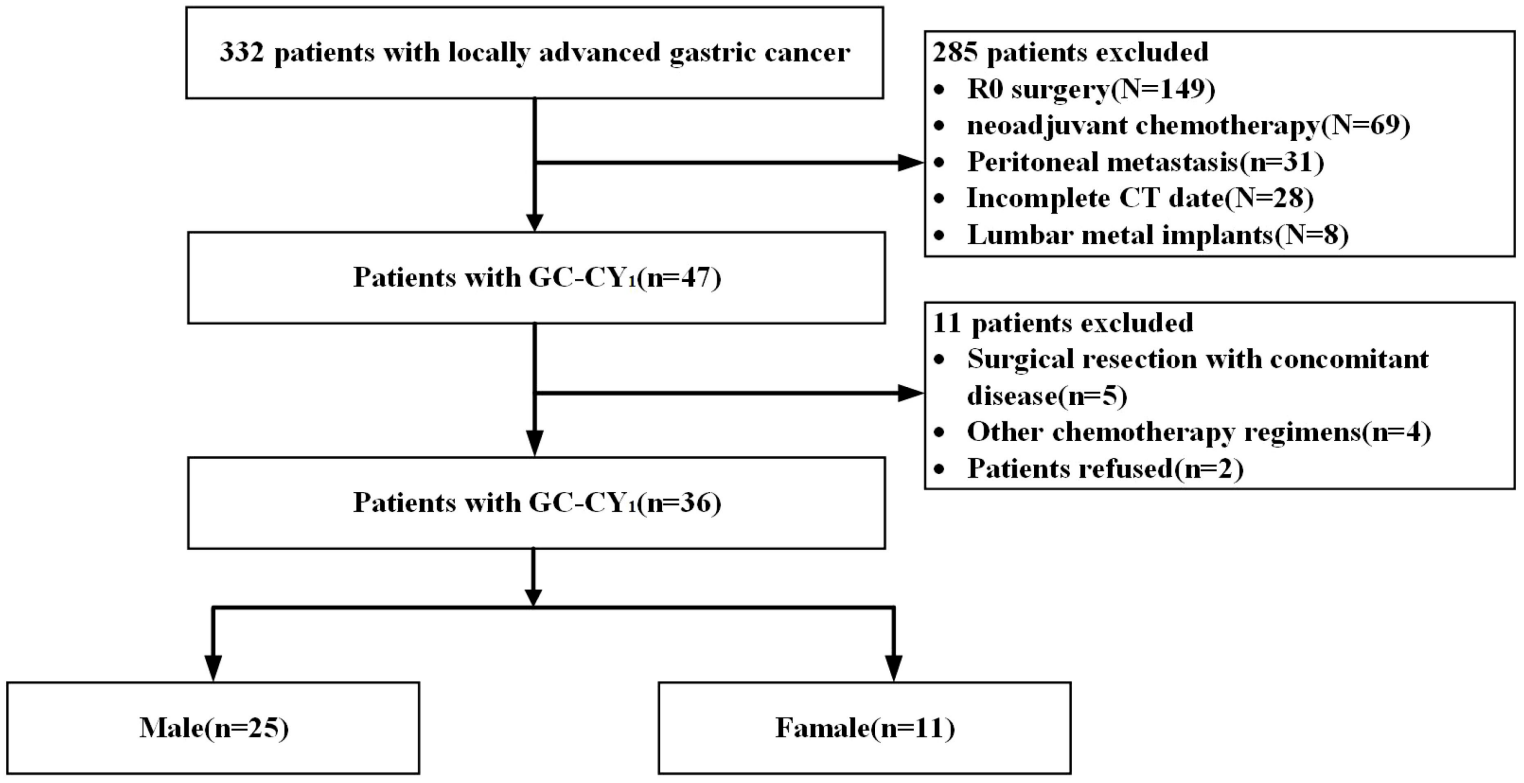
Flow chart of patient enrollment and exclusion.

**Figure 2:**
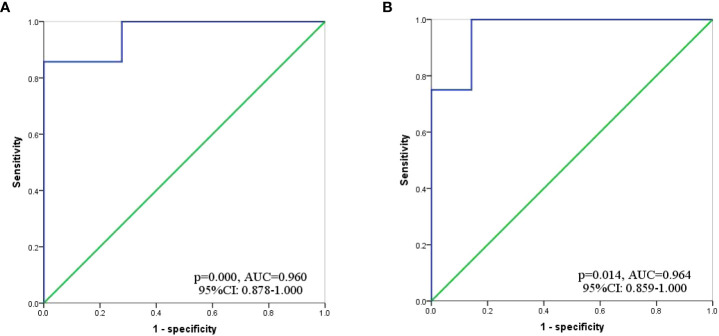
ROC curve to determine the optimal cut-off value for ΔSMI (%)/50 days. **(A)** Male; **(B)** Female.

Based on inclusion and exclusion criteria, we reviewed data of 36 GC-CY_1_ patients who received NIPS paclitaxel in combination with apatinib conversion therapy (**Figure 1**). Seven (19.44%) GC-CY_1_ patients were already in sarcopenia before conversion therapy, which was slightly more common in males (4/7, 57.14%). The proportion of sarcopenia (13/36, 36.11%) increased after conversion therapy, with 6 cases of newly developed sarcopenia and the remaining 7 patients with persistent sarcopenia. The mean ΔSMI (%)/50 days during conversion therapy was -6.35 ± 3.57% for the whole group of patients. The optimal cut-off value for ΔSMI (%)/50 days was not clearly defined, so ROC curves were used for clarification. The different ROC curves based on gender showed an area under the curve (AUC) value of 0.960 (95%CI: 0.878-1.000, p=0.000) for men and 0.964 (95%CI: 0.859-1.000, p=0.014) for women (**Figure 2**). According to the Youden’s index, the optimal cut-off value of male ΔSMI (%)/50 days was 9.53%, and that of female was 8.81%. The patients were grouped according to this optimal cut-off value, with 8 (22.22%) in the significant muscle loss (SML, male: ΔSMI (%)/50 days ≥ 9.53%; female: ΔSMI (%)/50 days ≥ 8.81%) group and 5 (62.50%) in the non-SML group.

### The relationship between sarcopenia and patient characteristics

**Figure 3:**
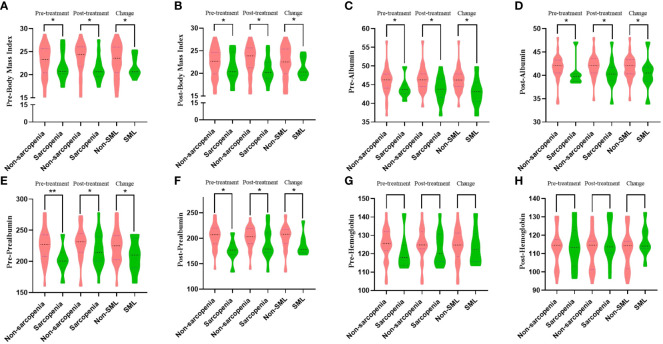
The relationship between sarcopenia and nutritional status outcomes before and after patient conversion therapy. SML: Significant muscle loss. **(A, B)** Relationship between sarcopenia during conversion therapy and body mass index before **(A)** and after **(B)** treatment. **(C, D)** Relationship between sarcopenia during conversion therapy and albumin before **(C)** and after **(D)** treatment. **(E, F)**: Relationship between sarcopenia during conversion therapy and prealbumin before **(E)** and after **(F)** treatment. **(G, H)** Relationship between sarcopenia during conversion therapy and haemoglobin before **(G)** and after **(H)** treatment. *p < 0.05, **p < 0.01.

The mean age of the whole group of patients was 56.3 ± 10.0 years, of which 25 (69.44%) cases were male. The relationship between the status of sarcopenia and nutrition-related outcomes before and after patient conversion therapy is summarised in **Figure 3**. The difference in BMI, albumin, and prealbumin between sarcopenic patients and non-sarcopenic patients before and after conversion therapy was more (all p<0.05). Meanwhile, patients who developed SML during conversion therapy had significantly lower levels of BMI (pre-: 21.83 ± 2.38 versus 23.06 ± 3.62, p=0.012; post-: 21.23 ± 2.28 versus 22.47 ± 3.45, p=0.036), albumin (pre-: 43.00 ± 3.97 versus 46.25 ± 3.25, p=0.011; post-: 40.35 ± 3.71 versus 41.66 ± 2.51, p=0.042) and prealbumin (pre-: 208.88 ± 26.98 versus 224.70 ± 26.41, p=0.004; post-: 186.73 ± 21.93 versus 200.97 ± 25.45, p=0.009) than those without significant muscle loss (non-SML), both pre- and post-treatment. In addition, there was no significant difference in haemoglobin levels between the SML group and the non-SML group before and after conversion treatment (pre-: 124.34 ± 8.97 versus 123.70 ± 9.69, p=0.862; post-: 112.29 ± 10.45 versus 116.73 ± 8.68, p=0.281). The relationship between all other clinicopathological features and the patient’s sarcopenia before and after conversion therapy is shown in **Table 1**.

**Table 1:** Patient and tumour characteristics (N = 36, %).

Characteristic	Overall (n=36)	Pre-treatment		p	Post-treatment		p	ΔSMI (%)/50 days		p
		Sarcopenia (n=7)	Non-sarcopenia (n=29)		Sarcopenia (n=13)	Non-sarcopenia (n=23)		SML (n=8)	Non-SML (n=28)	
**Age (years)**				1.000^*^			0.475^*^			1.000^*^
≤50	11 (30.56)	2 (28.57)	9 (31.03)		5 (38.46)	6 (26.09)		2 (25.00)	9 (32.14)	
>50	25 (69.44)	5 (71.43)	20 (68.97)		8 (61.54)	17 (73.91)		6 (75.00)	19 (67.86)	
**ECOG**				0.073^*^			0.016^*^			0.209^*^
0	30 (83.33)	4 (57.14)	26 (89.66)		8 (61.54)	22 (95.65)		5 (62.50)	25 (89.29)	
1	6 (16.67)	3 (42.86)	3 (10.34)		5 (38.46)	1 (4.35)		3 (37.50)	3 (10.71)	
**Gender**				0.650^*^			0.153^*^			0.961^*^
Male	25 (69.44)	4 (57.14)	21 (72.41)		7 (53.85)	18 (78.26)		5 (62.50)	20 (71.43)	
Female	11 (30.56)	3 (42.86)	8 (27.59)		6 (46.15)	5 (21.74)		3 (37.50)	8 (28.57)	
**Lesion site**				0.318^*^			0.067^*^			0.712^*^
Upper 1/3	13 (36.11)	1 (14.29)	12 (41.38)		1 (7.69)	12 (52.17)		2 (25.00)	11 (39.28)	
Middle 1/3	9 (25.00)	3 (42.86)	6 (20.69)		6 (46.15)	3 (13.04)		2 (25.00)	7 (25.00)	
Lower 1/3	14 (38.89)	3 (42.86)	11 (37.93)		6 (46.15)	8 (34.78)		4 (50.00)	10 (35.71)	
**Borrmann type**				1.000^*^			1.000^*^			1.000^*^
I–II	8 (22.22)	1 (14.29)	7 (24.13)		3 (23.08)	5 (21.74)		2 (25.00)	6 (21.43)	
III–IV	28 (77.78)	6 (85.71)	22 (75.86)		10 (76.92)	18 (78.26)		6 (75.00)	22 (78.57)	
**Histological**				0.573^*^			0.645^*^			0.858^*^
High-moderate	6 (16.67)	2 (28.57)	4 (13.79)		3 (23.08)	3 (13.04)		2 (25.00)	4 (14.29)	
Low	30 (83.33)	5 (71.43)	25 (86.21)		10 (76.92)	20 (86.96)		6 (75.00)	24 (85.71)	
**cT staging**				1.000^*^			1.000^*^			1.000^*^
T3	6 (16.67)	1 (14.29)	5 (17.24)		2 (15.38)	4 (17.39)		1 (12.50)	5 (17.86)	
T4	30 (83.33)	6 (85.71)	24 (82.76)		11 (84.62)	19 (82.61)		7 (87.50)	23 (82.14)	
**cN staging**				0.333^*^			0.693^*^			1.000^*^
N1–N2	9 (25.00)	3 (42.86)	6 (20.69)		4 (30.77)	5 (21.74)		2 (25.00)	7 (25.00)	
N3	27 (75.00)	4 (57.14)	23 (79.31)		9 (69.23)	18 (78.26)		6 (75.00)	21 (75.00)	
**Lesion size (cm)**				1.000^*^			1.000^*^			0.643^*^
<5	9 (25.00)	2 (28.57)	7 (24.13)		3 (23.08)	6 (26.09)		1 (12.50)	8 (28.57)	
≥5	27 (75.00)	5 (71.43)	22 (75.86)		10 (76.92)	17 (73.91)		7 (87.50)	20 (71.43)	

ECOG, Eastern Cooperative Oncology Group; BMI, body mass index; SML, Significant muscle loss. *Fisher’s exact test.

### The relationship between sarcopenia and treatment efficacy

**Figure 4:**
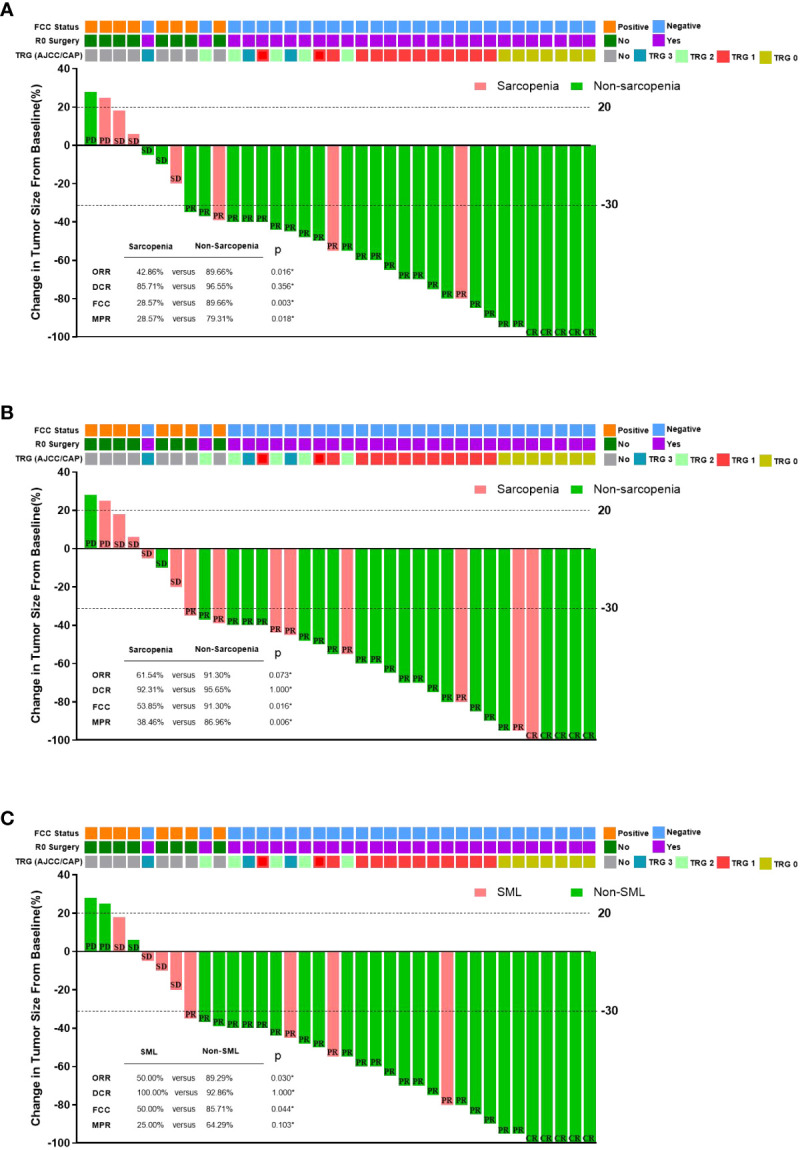
The relationship between sarcopenia and the efficacy of conversion therapy in GC-CY_1_ patients. **(A)** The relationship between pre-treatment sarcopenia and the efficacy of conversion therapy. **(B)** The relationship between post-treatment sarcopenia and the efficacy of conversion therapy. **(C)** The relationship between the presence of SML during treatment and the efficacy of conversion therapy. CR, complete remission; PR, partial response; SD, stable disease; PD, progressive disease; ORR, objective response rate; DCR, disease control rate; MPR, major pathologic response; FCC, free cancer cells; *Fisher’s exact test.

**Figure 4** summarises the relationship between sarcopenia and the efficacy of conversion therapy for GC-CY_1_ patients. All 36 GC-CY_1_ patients underwent abdominal CT scans after 3 cycles of NIPS paclitaxel combined with apatinib conversion therapy. Of 36 patients enrolled, 5 patients (13.89%) achieved CR, 24 patients achieved (66.67%) PR, and 5 patients (13.89%) achieved SD, while 2 patients (5.56%) developed PD, based on the evaluation by RECIST (version 1.1). Patients in the sarcopenic group had a lower ORR than the non-sarcopenic group both before (42.86% versus 89.66%, p=0.016) and after (61.54% versus 91.30%, p=0.073) treatment, this difference was also present in patients who developed SML versus those who did not during conversion therapy (50.00% versus 89.29%, p=0.030), but there was no difference in DCR between the groups (all p>0.05). Logistic univariate and multifactorial analyses identified pre-conversion therapy sarcopenia (OR=7.891, 95%CI: 1.984-24.156, p=0.008), the presence of SML during conversion therapy (OR=10.642, 95%CI: 2.676-43.531, p=0.002) and cT staging (OR=0.074, 95% CI: 0.009-0.659, p=0.022) were statistically significantly associated with achieving CR and PR in GC-CY_1_ patients (**Supplementary Table 1**).

All patients underwent laparoscopic exploration combined with abdominal exfoliative cytology, free cancer cells (FCC) were still present in the abdominal cavity in 8 patients (22.22%), while 28 patients (77.78%) who turned negative underwent R0 surgical resection. A subgroup analysis based on the presence or absence of sarcopenia before and after treatment found that all patients in the sarcopenic group had significantly lower rates of FCC conversion than those in the non-sarcopenic group (pre-: 28.57% versus 89.66%, p=0.003; post-: 53.85% versus 91.30%, p=0.016). In addition, patients who developed SML during conversion therapy had a lower rate of FCC conversion than those in the non-SML group (50.00% versus 85.71%, p=0.044). After univariate and multifactorial analyses, the presence of sarcopenia before conversion therapy (OR=8.923, 95%CI: 1.341-25.321, p=0.002) and the presence of SML during conversion therapy (OR=7.803, 95%CI: 1.106-16.189, p=0.001) were found to be the independent risk factors. (**Supplementary Table 2**).

According to the AJCC/CAP criteria for the assessment of pathological regression, seven (25.00%) of the 28 FCC-negative patients who underwent surgical resection achieved TRG grade 0, 13 patients (46.43%) achieved TRG grade 1, 5 patients (17.86%) were TRG grade 2, and 3 patients (10.71%) were TRG grade 3. We found that sarcopenia patients had lower MPR than normal groups before conversion therapy (28.57% vs. 79.31%, p=0.018) and those who developed sarcopenia after conversion therapy (38.46% vs. 86.96%, p=0.006), but there was no significant difference between patients with SML only during conversion therapy (25.00% vs. 64.29%, p=0.103) and patients without SML. Logistic univariate and multifactorial analyses revealed that the presence of non-Sarcopenic after conversion therapy (OR=0.007, 95%CI: 0.004-0.224, p=0.006) and histologically moderate to high differentiation (OR=0.014, 95%CI: 0.002-0.412, p=0.010) were independent protective factors influencing whether GC-CY_1_ patients were graded as TRG0 and TRG1 for pathological regression (**Supplementary Table 3**).

### The relationship between sarcopenia and adverse effects

Among the 36 GC-CY_1_ patients who received conversion therapy, the incidence of hematological toxicity of grade III and above was 19.44%(7/36) (**Table 2**). We observed that patients with sarcopenia before treatment were more likely to have grade≥3 neutropenia(40.00% vs. 0, p=0.033) and nausea/vomiting(40.00% vs. 0, p=0.033) than patients without sarcopenia. This difference did not appear in patients with post-treatment sarcopenia and non-sarcopenia. More importantly, patients with SML experienced a significant increase in grade ≥3 toxicity during conversion therapy compared to patients without SML, particularly in leukopenia(33.33% vs. 0, p=0.044), neutropenia(33.33% vs. 0, p=0.044), and nausea/vomiting(33.33% vs. 0, p=0.044).

**Table 2:** Relationship between sarcopenia and adverse effects during conversion therapy in GC-CY_1_ patients.

Adverse effects	Pre-treatment		p	Post-treatment		p	ΔSMI (%)/50 days			p
	Sarcopenia(n=7)	No-sarcopenia(n=29)		Sarcopenia(n=13)	No-sarcopenia(n=23)		SML(n=8)		No-SML(n=28)	
**Leukopenia**										
Yes/No	3/4	5/24	0.167*	5/8	3/20	0.107*	6/2	2/26		<0.001*
≥3 grade/<3grade	2/5	0/29	0.033*	2/13	0/23	0.149*	2/6	0/28		0.044*
**Neutropenia**										
Yes/No	4/3	3/26	0.016*	4/9	3/20	0.225*	5/3	2/26		0.003*
≥3 grade/<3grade	2/5	0/29	0.033*	1/12	1/22	1.000*	2/6	0/28		0.044*
**Thrombocytopenia**										
Yes/No	3/4	1/28	0.018*	4/9	0/23	0.012*	4/4	0/28		0.001*
≥3 grade/<3grade	0/7	0/29	1.000*	0/13	0/23	1.000*	0/8	0/28		1.000*
**Decreased hemoglobin**										
Yes/No	1/6	0/29	0.194*	1/12	0/23	0.361*	1/7	0/28		0.222*
≥3 grade/<3grade	0/7	0/29	1.000*	0/13	0/23	1.000*	0/8	0/28		1.000*
**Nausea/vomiting**										
Yes/No	5/2	4/25	0.006*	5/8	4/19	0.235*	7/1	2/26		<0.001*
≥3 grade/<3grade	2/5	0/29	0.033*	1/12	1/22	1.000*	2/6	0/28		0.044*
**Diarrhea**										
Yes/No	1/6	0/29	0.194*	1/12	0/23	0.361*	1/7	0/28		0.222*
≥3 grade/<3grade	0/7	0/29	1.000*	0/13	0/23	1.000*	0/8	0/28		1.000*
**Anorexia**										
Yes/No	2/5	1/28	0.090*	3/10	0/23	0.040*	3/5	0/28		0.008*
≥3 grade/<3grade	0/7	0/29	1.000*	0/13	0/23	1.000*	0/8	0/28		1.000*
**Peripheral sensory neuropathy**										
Yes/No	1/6	0/29	0.194*	1/12	0/23	0.361*	1/7	0/28		0.222*
≥3 grade/<3grade	0/7	0/29	1.000*	0/13	0/23	1.000*	0/8	0/28		1.000*
**AST/ALT increased**										
Yes/No	0/7	1/28	1.000*	1/12	0/23	0.361*	0/8	1/27		1.000*
≥3 grade/<3grade	0/7	0/29	1.000*	0/13	0/23	1.000*	0/8	0/28		1.000*
**Proteinuria**										
Yes/No	2/5	2/27	0.163*	3/10	1/22	0.124*	2/6	2/26		0.207*
≥3 grade/<3grade	0/7	0/29	1.000*	0/13	0/23	1.000*	0/8	0/28		1.000*
**Oral mucositis**										
Yes/No	1/6	0/29	0.194*	0/13	1/22	1.000*	1/7	0/28		0.222*
≥3 grade/<3grade	0/7	0/29	1.000*	0/13	0/23	1.000*	0/8	0/28		1.000*
**Fatigue**										
Yes/No	2/5	1/28	0.090*	3/10	0/23	0.040*	3/5	0/28		0.008*
≥3 grade/<3grade	1/6	0/29	0.194*	1/12	0/23	0.361*	1/7	0/28		0.222*

According to Common Terminology Criteria for Adverse Events (version 3.0). AST, aspartate transaminase; ALT, alanine aminotransferase. *Fisher’s exact test.

### The relationship between sarcopenia and prognosis

**Figure 5:**
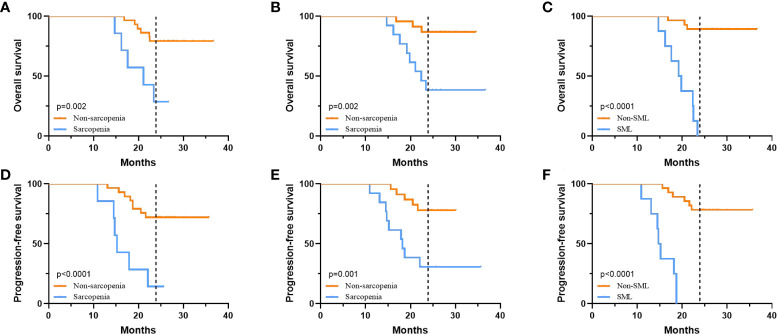
The relationship between sarcopenia and prognosis. **(A)** 2-year OS of patients in the pre-treatment sarcopenic group versus the non-sarcopenic group. **(B)** 2-year OS of patients in the post-treatment sarcopenic group versus the non-sarcopenic group. **(C)** 2-year OS for patients in the SML group versus the non-SML group who developed SML during treatment. **(D)** 2-year PFS of patients in the pre-treatment sarcopenic group versus the non-sarcopenic group. **(E)** 2-year PFS of patients in the post-treatment sarcopenic group versus the non-sarcopenic group. **(F)** 2-year PFS for patients in the SML group versus the non-SML group who developed SML during treatment. SML, significant muscle loss.

The median follow-up time for all patients was 25.5 (15.6-38.4) months, with 2-year OS and PFS rates of 69.44% and 58.33%, respectively. **Figure 5** summarises the 2-year OS and PFS for GC-CY_1_ patients. The 2-year OS (pre: 28.57% versus 79.31%, p=0.002; post: 38.46% versus 86.96%, p=0.002) and PFS (pre: 14.29% versus 72.41%, p<0.001; post: 30.77% versus 78.26%, p=0.001) rates were significantly different between the pre- and post-treatment sarcopenia and non-sarcopenia groups. In addition, 2-year OS (0% versus 89.29%, p<0.001) and PFS (0% versus 78.57%, p<0.001) were significantly lower in the SML group than in the non-SML group during conversion therapy. Cox multifactorial analysis found that status of FCC (OS: HR=5.164, 95%CI: 2.146-14.709, p=0.011; PFS: HR=5.092, 95%CI: 2.112-17.809, p=0.012), pre-treatment sarcopenia (OS: HR=6.341, 95%CI: 1.269-18.943, p=0.001; PFS: HR=8.212, 95%CI: 1.569-36.582, p=0.001), newly developed sarcopenia after conversion therapy (OS: HR=3.189, 95%CI: 1.023-9.811, p=0.012; PFS: HR=3.084, 95%CI: 1.042-14.236, p=0.013) and the presence of SML during treatment (OS: HR=10.234, 95%CI: 2.532-54.231, p=0.002; PFS: HR=9.562, 95%CI: 2.341-38.092, p=0.002) were all independent risk factors for 2 year OS and PFS in GC-CY_1_ patients **(** **Tables 3**, **4** **)**.

**Table 3:** Univariable and multifactorial analysis for overall survival (N = 36).

Variable	Univariable Analysis		Multivariable Analysis	
	HR (95% CI)	p	HR (95% CI)	p
**Age (years)**		0.351		
≤50	Reference			
>50	0.490 (0.137–1.755)			
**ECOG**		0.235		
1	Reference			
0	0.457 (0.086–2.433)			
**Gender**		0.619		
Male	Reference			
Female	0.733 (0.201–2.678)			
**Lesion site**				
Upper 1/3	Reference			
Middle 1/3	0.726 (0.035–2.155)	0.245		
Lower 1/3	0.632 (0.042–1.232)	0.361		
**Pre-BMI(Kg/m^2^)**		0.764		
Underweight (<18.5)	Reference			
Normal (≥18.5)	0.731 (0.119–4.499)			
**Post-BMI(Kg/m^2^)**		0.978		
Underweight (<18.5)	Reference			
Normal (≥18.5)	0.979 (0.209–4.578)			
**Borrmann type**		0.792		
III–IV	Reference			
I–II	0.814 (0.193–3.435)			
**Histological**		0.737		
Low	Reference			
High-moderate	0.770 (0.144–4.103)			
**cT staging**		0.445		
T4	Reference			
T3	0.458 (0.098–2.136)			
**cN staging**		0.539		
N3	Reference			
N1–N2	0.622 (0.163–2.378)			
**Lesion size(cm)**		0.574		
≥5	Reference			
<5	0.647 (0.167–2.505)			
**FCC**		0.001		0.011
Negative	Reference		Reference	
Positive	5.617 (1.190–26.510)		5.164 (2.146–14.709)	
**Sarcopenia**				
Non-Sarcopenic	Reference		Reference	
Pre-treatment Sarcopenia	8.043 (1.280–50.520)	0.001	6.341 (1.269-18.943)	0.001
Newly developed Sarcopenia	4.740 (0.575-39.090)	0.035	3.189 (1.023-9.811)	0.012
**ΔSMI (%)/50 days**		0.000		0.002
No-SML	Reference		Reference	
SML	17.031 (3.028–95.840)		10.234 (2.532–54.231)	

ECOG, Eastern Cooperative Oncology Group; SML, Significant muscle loss; FCC, Free cancer cells.

**Table 4:** Univariable and multifactorial analysis for progression-free survival (N = 36).

Variable	Univariable Analysis		Multivariable Analysis	
	HR (95% CI)	p	HR (95% CI)	p
**Age (years)**		0.709		
≤50	Reference			
>50	0.803 (0.264–2.441)			
**ECOG**		0.125		
1	Reference			
0	0.370 (0.061–2.253)			
**Gender**		0.592		
Male	Reference			
Female	0.743 (0.235–2.352)			
**Lesion site**				
Upper 1/3	Reference			
Middle 1/3	0.792 (0.123–2.345)	0.283		
Lower 1/3	0.526 (0.067–2.545)	0.326		
**Pre-BMI(Kg/m^2^)**		0.563		
Underweight (<18.5)	Reference			
Normal (≥18.5)	0.554 (0.112–2.750)			
**Post-BMI(Kg/m^2^)**		0.703		
Underweight (<18.5)	Reference			
Normal (≥18.5)	0.749 (0.193–2.910)			
**Borrmann type**		0.410		
III–IV	Reference			
I–II	0.619 (0.166–2.301)			
**Histological**		0.471		
Low	Reference			
High-moderate	0.643 (0.148–2.797)			
**cT staging**		0.823		
T4	Reference			
T3	0.844 (0.206–3.462)			
**cN staging**		0.776		
N3	Reference			
N1–N2	0.832 (0.247–2.802)			
**Lesion size(cm)**		0.918		
≥5	Reference			
<5	0.866 (0.261–2.870)			
**FCC**		0.000		0.012
Negative	Reference		Reference	
Positive	6.155 (1.359–27.890)		5.092 (2.112–17.809)	
**Sarcopenia**				
Non-Sarcopenic	Reference		Reference	
Pre-treatment Sarcopenia	7.539 (1.354–41.980)	<0.0001	8.212 (1.569–36.582)	0.001
Newly developed Sarcopenia	2.939 (1.464-18.630)	0.019	3.084 (1.042-14.236)	0.013
**ΔSMI (%)/50 days**		0.000		0.002
No-SML	Reference		Reference	
SML	11.710 (2.078–66.030)		9.562 (2.341–38.092)	

ECOG, Eastern Cooperative Oncology Group; SML, Significant muscle loss; FCC, Free cancer cells.

## Discussion

This study retrospectively analysed the effect of sarcopenia and skeletal muscle loss on clinical outcomes among 36 patients with GC-CY_1_, and they were included in a registered prospective clinical study (NCT03718624) during NIPS paclitaxel in combination with apatinib conversion therapy. Firstly, we observed that 19.44% of patients had sarcopenia before conversion therapy and this rose to 36.11% after conversion therapy. Secondly, SML was defined as above 9.53% of skeletal muscle loss during conversion therapy (ΔSMI (%)/50 days) for male patients and above 8.81% for females, with only 22.22% of all patients experiencing SML during conversion therapy. Thirdly, there was a strong correlation between sarcopenia and changes in laboratory indicators related to nutritional status during conversion therapy. Finally, sarcopenia before and after conversion therapy and the presence of SML during conversion therapy were significantly associated with both the patient’s immediate outcome and long-term prognosis.

In previous studies, BMI has been used to assess the occurrence of postoperative complications and the long-term prognosis of patients with malignancies (23). Nevertheless, growing evidence has found that using a single indicator of BMI alone for assessment is less effective because it does not take into account differences in gender and body composition, and in particular, it ignores changes in muscle (24). Presently, abdominal CT is widely used for GC-CY_1_ patients who require it before and after conversion therapy to assess treatment efficacy (17, 18). Moreover, there is growing attention to abdominal CT based body composition analysis, particularly at the L3 level (25, 26). Several studies have confirmed that skeletal muscle measurements at the L3 level have a good ability to predict postoperative complications, tumour response and prognosis in patients with malignancies (26, 27). A prospective study of 225 patients with foregut cancer found that 23.9% of patients had SML after preoperative neoadjuvant chemotherapy, with the highest proportion of patients with oesophageal and gastric cancers (28). As for GC-CY_1_ patients, our results found SML in 22.22% of patients, which is consistent with the results of previous studies (28, 29), it indicates that GC-CY_1_ patients also experience SML in conversion therapy.

A retrospective study involved 31 patients with oesophageal cancer who received neoadjuvant chemotherapy and underwent surgery found that pathological regression responses (grade 2 or higher) were more common in the non-muscle reduced group than in the muscle reduced group (53.3% vs. 25.0%) (30). However, a retrospective study by Takeda T et al. reached contradictory conclusions (31). In gastric cancer, few reports have evaluated the effect of sarcopenia on the efficacy of chemotherapy. For GC-CY_1_, our study revealed that sarcopenia before conversion therapy and SML development during therapy were strongly associated with local efficacy response and were independent risk factors for the success of conversion therapy. The reasons for the differences between the results of this study and previous studies may relate to treatment regimens, ethnicity of subjects, muscle mass measurements and the selection of optimal cut-off value. Therefore, a multicentre prospective study with large sample size is needed to validate the association between sarcopenia and treatment efficacy before and after conversion therapy treatment.

Currently, the association between sarcopenia and OS in patients with advanced gastric cancer are inconsistent, some studies report worse OS in patients with sarcopenia before or after treatment than in those without sarcopenia (25, 26), while others report that they do not have any association (26, 29). In this study, we performed Cox multivariate analysis of OS and PFS to explore the relationship between sarcopenia before and after conversion therapy and skeletal muscle changes during conversion therapy and prognosis in GC-CY_1_ patients. We found that pre-treatment sarcopenia, newly developed sarcopenia after conversion therapy and the presence of SML during treatment were independent risk factors for OS and PFS in GC-CY_1_ patients and were not associated with sarcopenia before treatment, which is consistent with previous findings on body composition (25, 32). The mechanisms by which sarcopenia affects the long-term prognosis of GC-CY_1_ patients are currently unknown and the reasons may be multifaceted. Firstly, gastric cancer is a highly aggressive malignancy, accompanied by higher catabolic and basal metabolic rates (33). The high metabolism aggravates systemic inflammatory response and causes severe muscle wastage, which ultimately leads to the development of sarcopenia (33). Furthermore, muscular sarcopenia exacerbates the inflammatory response of the body, which in turn is closely related to the long-term survival of the patient, resulting in a series of chain reactions leading to a poor prognosis (34).

Certain limitations of this study merit consideration. Firstly, this study is a single-centre, small sample prospective study and there may be some selection bias in the study population. Secondly, this study only examined the impact of skeletal muscle changes at two time points on treatment efficacy, pathological regression response and prognosis, without further in-depth analysis of skeletal muscle changes following later treatment. Thirdly, we did not investigate adverse effects during conversion therapy with NIPS paclitaxel plus apatinib. The presence of adverse effects, particularly gastrointestinal reactions, can affect patients’ nutritional status and further affect muscle loss. Therefore, the relationship between sarcopenia and adverse drug reactions during neoadjuvant therapy is required for further analyses in subsequent studies. Finally, further studies are needed to investigate the mechanisms of skeletal muscle loss and to find how to preserve skeletal muscle mass during conversion therapy to improve clinical outcomes.

## Conclusions

In conclusion, although the average change in the SMI during conversion therapy in GC-CY_1_ patients was not significant, skeletal muscle loss(SML, male:ΔSMI (%)/50 days≥9.53%; female:ΔSMI(%)/50 days≥8.81%)was independently associated with poor OS and PFS. Meanwhile, pre-treatment sarcopenia and new sarcopenia after conversion therapy are also intimately associated with the success of conventional treatment and are independent risk factors for patient prognosis.

## Data availability statement

The raw data supporting the conclusions of this article will be made available by the authors, without undue reservation.

## Ethics statement

This trial was approved by the Ethics Committee of the Fourth Hospital of Hebei Medical University (approval number: 2018088). The patients/participants provided their written informed consent to participate in this study.

## Author contributions

(I) Conception and design: QZ; (II) Administrative support: QZ; (III) Provision of study materials or patients: P’AD, PY, YT, HG; (IV) Collection and assembly of data: P’AD, PY, YT, and HG; (V) Data analysis and interpretation: P’AD and CS; (VI) Manuscript writing: All authors; (VII) Final approval of manuscript: All authors.

## Funding

This work was supported by the Cultivating Outstanding Talents Project of Hebei Provincial Government Fund (No.2019012); Hebei public health committee county-level public hospitals suitable health technology promotion and storage project (No.2019024); Hebei University Science and Technology Research Project (No.ZD2019139).

## Conflict of interest

All authors have completed the ICMJE uniform disclosure form.

The authors declare that the research was conducted in the absence of any commercial or financial relationships that could be construed as a potential conflict of interest.

## Publisher’s note

All claims expressed in this article are solely those of the authors and do not necessarily represent those of their affiliated organizations, or those of the publisher, the editors and the reviewers. Any product that may be evaluated in this article, or claim that may be made by its manufacturer, is not guaranteed or endorsed by the publisher.
