# Supplementary material for: Impact of skeletal muscle loss during conversion therapy on clinical outcomes in lavage cytology positive patients with gastric cancer
Source: Front Oncol. 2022 Sep 23;12:949511. doi: 10.3389/fonc.2022.949511 (PMC9615921; doi:10.3389/fonc.2022.949511)
Supplement: Supplementary file 1 [file DataSheet_1.docx]

**[Supplementary](https://www.frontiersin.org/articles/10.3389/fnut.2022.796601/full#SM2)Table 1. Logistic univariate and multifactorial analysis of risk factors affecting whether GC-CY_1_ patients achieve CR and PR(N=36).**

| **Variable** | **Univariable Analysis** | |  | **Multivariable Analysis** | |
| --- | --- | --- | --- | --- | --- |
|  | **OR (95% CI)** | **p** |  | **OR (95% CI)** | **p** |
| **Age (years)** |  | 0.139 |  |  |  |
| ≤50 | Reference |  |  |  |  |
| >50 | 0.107(0.006–2.061) |  |  |  |  |
| **ECOG** |  | 0.351 |  |  |  |
| 1 | Reference |  |  |  |  |
| 0 | 0.241(0.012–4.801) |  |  |  |  |
| **Gender** |  | 0.436 |  |  |  |
| Male | Reference |  |  |  |  |
| Female | 0.508(0.092–2.792) |  |  |  |  |
| **Lesion site** |  |  |  |  |  |
| Upper 1/3 | Reference |  |  |  |  |
| Middle 1/3 | 0.676(0.050–3.250) | 0.371 |  |  |  |
| Lower 1/3 | 0.516(0.312–2.589) | 0.157 |  |  |  |
| **Pre-BMI(Kg/m^2^)** |  | 0.767 |  |  |  |
| Underweight (<18.5) | Reference |  |  |  |  |
| Normal (≥18.5) | 0.692(0.061–7.872) |  |  |  |  |
| **Post-BMI(Kg/m^2^)** |  | 0.357 |  |  |  |
| Underweight (<18.5) | Reference |  |  |  |  |
| Normal (≥18.5) | 0.400(0.057–2.812) |  |  |  |  |
| **Borrmann type** |  | 0.579 |  |  |  |
| III–IV | Reference |  |  |  |  |
| I–II | 0.524(0.054–5.129) |  |  |  |  |
| **Histological** |  | 0.703 |  |  |  |
| Low | Reference |  |  |  |  |
| High-moderate | 0.634(0.064–6.371) |  |  |  |  |
| **cT staging** |  | 0.021 |  |  | 0.022 |
| T4 | Reference |  |  | Reference |  |
| T3 | 0.153(0.078–0.490) |  |  | 0.074(0.009–0.659) |  |
| **cN staging** |  | 0.703 |  |  |  |
| N3 | Reference |  |  |  |  |
| N1–N2 | 0.639(0.064–6.371) |  |  |  |  |
| **Lesion size(cm)** |  | 0.475 |  |  |  |
| ≥5 | Reference |  |  |  |  |
| <5 | 0.438(0.045–4.227) |  |  |  |  |
| **Pre-treatment sarcopenia** |  | 0.010 |  |  | 0.008 |
| No | Reference |  |  | Reference |  |
| Yes | 12.000(1.791–80.389) |  |  | 7.891(1.984–24.156) |  |
| **Post-treatment sarcopenia** |  | 0.096 |  |  |  |
| No | Reference |  |  |  |  |
| Yes | 12.27(0.641–234.892) |  |  |  |  |
| **ΔSMI (%)/50 days** |  | 0.023 |  |  | 0.002 |
| No-SML | Reference |  |  | Reference |  |
| SML | 8.333(1.335–52.041) |  |  | 10.642(2.676–43.531) |  |

Abbreviations: ECOG, Eastern Cooperative Oncology Group; SML: Significant muscle loss.

[**Supplementary**](https://www.frontiersin.org/articles/10.3389/fnut.2022.796601/full#SM2)**Table 2. Logistic univariate and multifactorial analysis of risk factors affecting whether GC-CY_1_ patients are negative for FCC(N=36).**

| **Variable** | **Univariable Analysis** | |  | **Multivariable Analysis** | |
| --- | --- | --- | --- | --- | --- |
|  | **OR (95% CI)** | **p** |  | **OR (95% CI)** | **p** |
| **Age (years)** |  | 0.233 |  |  |  |
| ≤50 | Reference |  |  |  |  |
| >50 | 0.257(0.028–2.400) |  |  |  |  |
| **ECOG** |  | 0.091 |  |  |  |
| 1 | Reference |  |  |  |  |
| 0 | 0.200(0.031–1.293) |  |  |  |  |
| **Gender** |  | 0.186 |  |  |  |
| Male | Reference |  |  |  |  |
| Female | 0.333(0.065–1.700) |  |  |  |  |
| **Lesion site** |  |  |  |  |  |
| Upper 1/3 | Reference |  |  |  |  |
| Middle 1/3 | 0.536(0.041–3.528) | 0.214 |  |  |  |
| Lower 1/3 | 0.426(0.112–2.421) | 0.316 |  |  |  |
| **Pre-BMI(Kg/m^2^)** |  | 0.054 |  |  |  |
| Underweight (<18.5) | Reference |  |  |  |  |
| Normal (≥18.5) | 0.093(0.008–1.043) |  |  |  |  |
| **Post-BMI(Kg/m^2^)** |  | 0.032 |  |  | 0.181 |
| Underweight (<18.5) | Reference |  |  | Reference |  |
| Normal (≥18.5) | 0.076(0.007–0.797) |  |  | 0.782(0.121–5.231) |  |
| **Borrmann type** |  | 0.831 |  |  |  |
| III–IV | Reference |  |  |  |  |
| I–II | 0.818(0.130–5.138) |  |  |  |  |
| **Histological** |  | 0.296 |  |  |  |
| Low | Reference |  |  |  |  |
| High-moderate | 0.204(0.010–4.02) |  |  |  |  |
| **cT staging** |  | 0.479 |  |  |  |
| T4 | Reference |  |  |  |  |
| T3 | 0.500(0.073–3.406) |  |  |  |  |
| **cN staging** |  | 0.361 |  |  |  |
| N3 | Reference |  |  |  |  |
| N1–N2 | 0.455(0.084–2.469) |  |  |  |  |
| **Lesion size(cm)** |  | 0.370 |  |  |  |
| ≥5 | Reference |  |  |  |  |
| <5 | 0.357(0.038–3.389) |  |  |  |  |
| **Pre-treatment sarcopenia** |  | 0.021 |  |  | 0.002 |
| No | Reference |  |  | Reference |  |
| Yes | 7.667(1.363–43.136) |  |  | 8.923(1.341–25.321) |  |
| **Post-treatment sarcopenia** |  | 0.073 |  |  |  |
| No | Reference |  |  |  |  |
| Yes | 14.807(0.780–281.219) |  |  |  |  |
| **ΔSMI (%)/50 days** |  | 0.044 |  |  | 0.001 |
| No-SML | Reference |  |  | Reference |  |
| SML | 6.000(1.049–34.318) |  |  | 7.803(1.106–16.189) |  |

Abbreviations: ECOG, Eastern Cooperative Oncology Group; SML: Significant muscle loss.

[**Supplementary**](https://www.frontiersin.org/articles/10.3389/fnut.2022.796601/full#SM2)**Table 3. Logistic univariate and multifactorial analysis of risk factors affecting whether GC-CY_1_ patients were graded as TRG0 and TRG1 for pathological regression(N=28).**

| **Variable** | **Univariable Analysis** | |  | **Multivariable Analysis** | |
| --- | --- | --- | --- | --- | --- |
|  | **OR (95% CI)** | **p** |  | **OR (95% CI)** | **p** |
| **Age (years)** |  | 0.324 |  |  |  |
| ≤50 | Reference |  |  |  |  |
| >50 | 0.429(0.080–2.309) |  |  |  |  |
| **ECOG** |  | 0.159 |  |  |  |
| 1 | Reference |  |  |  |  |
| 0 | 0.158(0.012–2.063) |  |  |  |  |
| **Gender** |  | 0.341 |  |  |  |
| Male | Reference |  |  |  |  |
| Female | 0.417(0.069–2.527) |  |  |  |  |
| **Lesion site** |  |  |  |  |  |
| Upper 1/3 | Reference |  |  |  |  |
| Middle 1/3 | 0.823(0.152–3.23) | 0.511 |  |  |  |
| Lower 1/3 | 0.416(0.223–2.512) | 0.626 |  |  |  |
| **Pre-BMI(Kg/m^2^)** |  | 0.435 |  |  |  |
| Underweight (<18.5) | Reference |  |  |  |  |
| Normal (≥18.5) | 0.294(0.014–6.364) |  |  |  |  |
| **Post-BMI(Kg/m^2^)** |  | 0.537 |  |  |  |
| Underweight (<18.5) | Reference |  |  |  |  |
| Normal (≥18.5) | 0.529(0.071–3.978) |  |  |  |  |
| **Borrmann type** |  | 0.203 |  |  |  |
| III–IV | Reference |  |  |  |  |
| I–II | 0.294(0.045–1.938) |  |  |  |  |
| **Histological** |  | 0.018 |  |  | 0.010 |
| Low | Reference |  |  | Reference |  |
| High-moderate | 0.026(0.001–0.529) |  |  | 0.014(0.002–0.412) |  |
| **cT staging** |  | 0.320 |  |  |  |
| T4 | Reference |  |  |  |  |
| T3 | 0.333(0.038–2.910) |  |  |  |  |
| **cN staging** |  | 0.475 |  |  |  |
| N3 | Reference |  |  |  |  |
| N1–N2 | 0.429(0.042–4.392) |  |  |  |  |
| **Lesion size(cm)** |  | 0.256 |  |  |  |
| ≥5 | Reference |  |  |  |  |
| <5 | 0.265(0.027–2.625) |  |  |  |  |
| **Pre-treatment sarcopenia** |  | 0.073 |  |  |  |
| Yes | Reference |  |  |  |  |
| No | 0.529(0.071–3.978) |  |  |  |  |
| **Post-treatment sarcopenia** |  | 0.014 |  |  | 0.006 |
| Yes | Reference |  |  | Reference |  |
| No | 0.027(0.004–0.169) |  |  | 0.007(0.004–0.224) |  |
| **ΔSMI (%)/50 days** |  | 0.103 |  |  |  |
| SML | Reference |  |  |  |  |
| No-SML | 0.333(0.038–2.910) |  |  |  |  |

Abbreviations: ECOG, Eastern Cooperative Oncology Group; SML: Significant muscle loss.
